# Retinal Pigment Epithelium Remodeling in Mouse Models of Retinitis Pigmentosa

**DOI:** 10.3390/ijms22105381

**Published:** 2021-05-20

**Authors:** Debora Napoli, Martina Biagioni, Federico Billeri, Beatrice Di Marco, Noemi Orsini, Elena Novelli, Enrica Strettoi

**Affiliations:** 1 CNR Neuroscience Institute, 56124 Pisa, Italy; martina.biagioni@humanitasresearch.it (M.B.); federicobilleri@gmail.com (F.B.); beatrice.dimarco@in.cnr.it (B.D.M.); noemi.orsini@in.cnr.it (N.O.); elena.novelli@in.cnr.it (E.N.); 2 Department of Biology, University of Pisa, 56126 Pisa, Italy; 3 Regional Doctorate School in Neuroscience, Universities of Florence, Pisa and Siena, 50139 Florence, Italy

**Keywords:** retinitis pigmentosa, retinal pigment epithelium, rd9 mouse, rd10 mouse, Tvrm4 mouse, blood retinal barrier, zonula occludens, immunocytochemistry, dextranes

## Abstract

In retinitis pigmentosa (RP), one of many possible genetic mutations causes rod degeneration, followed by cone secondary death leading to blindness. Accumulating evidence indicates that rod death triggers multiple, non-cell-autonomous processes, which include oxidative stress and inflammation/immune responses, all contributing to cone demise. Inflammation relies on local microglia and recruitment of immune cells, reaching the retina through breakdowns of the inner blood retinal barrier (iBRB). Leakage in the inner retina vasculature suggests similarly altered outer BRB, formed by junctions between retinal pigment epithelium (RPE) cells, which are crucial for retinal homeostasis, immune response, and privilege. We investigated the RPE structural integrity in three models of RP (rd9, rd10, and Tvrm4 mice) by immunostaining for zonula occludens-1 (ZO-1), an essential regulatory component of tight junctions. Quantitative image analysis demonstrated discontinuities in ZO-1 profiles in all mutants, despite different degrees of photoreceptor loss. ZO-1 interruption zones corresponded to leakage of in vivo administered, fluorescent dextran through the choroid-RPE interface, demonstrating barrier dysfunction. Dexamethasone, administered to rd10 mice for rescuing cones, also rescued RPE structure. Thus, previously undetected, stereotyped abnormalities occur in the RPE of RP mice; pharmacological targeting of inflammation supports a feedback loop leading to simultaneous protection of cones and the RPE.

## 1. Introduction

The retinal pigment epithelium (RPE) is a monolayer of polygonal cells located between the photoreceptors and the choriocapillaris, delimited by Bruch’s membrane. A close spatial relationship between photoreceptors and RPE cells is common to the light-sensitive organs of both invertebrate and vertebrate animals, and proper photoreceptor physiology depends on interactions between photosensitive and pigmented cells. The regularly tiled RPE cells, typically of hexagonal shape, perform a variety of functions, all relevant for retinal physiology and homeostasis. The RPE participates in the visual cycle by recycling retinoids, fundamental for phototransduction; melanin granules absorb excess light, ameliorating vision and reducing photo-oxidative stress by scavenging light-induced free radicals. The RPE delivers nutrients and phagocytes photoreceptor outer segments, also disposing and recycling waste products. The RPE is also involved in the production and secretion of growth factors, controls the retinal immune response and privilege, and finally, its tight junction complexes are the main constituents of the outer retinal blood barrier (BRB), whose integrity is necessary to protect the retina from exogenous insults and excessive inflammation [1]. 

Converging evidence shows that dysfunctions of the RPE of various origin contribute to the pathogenesis of age-related macular degeneration (AMD), a leading cause of blindness in the elderly in industrialized countries. A large fraction of the scientific literature on the RPE is dedicated to study this organ in conjunction with its implications in AMD and for the possibility to develop therapeutic options [2,3,4,5]. Existing studies range from the molecular to clinical level and rely on cellular and animal models, as well as on clinical observations, altogether supporting the notions of a multifactorial nature of AMD with a specific contribution of (among others) oxidative stress and mitochondrial dysfunction [6,7]. In addition, the notion that the RPE can interact with the immune system has gained increasing attention in virtue of the finding that various forms of retinal degeneration (including AMD) are associated with development of inflammation and secondary immune reaction or with an insufficiency to silence it. This is in agreement with the proven influence of sustained immune responses occurring in other neurodegenerative conditions, such as Alzheimer’s disease [8]. 

Correspondingly, fewer studies (but see, for instance, [9,10]) are dedicated to elucidating the fine molecular and structural alterations of the RPE in retinitis pigmentosa (RP), a rare and yet incurable family of diseases of genetic origin, in which migration of melanin granules and cells from the RPE to the neural retina explains the name of the pathology, without, however, investigating the possible pathogenic role of RPE abnormalities, or addressing the options to explore this layer as a therapeutic target. Indeed, 18^th^ century, now classical studies, report how the term “retinitis pigmentosa tends to exalt the one term of the disease at the expense of the other”, exposing the difficulty to attribute the correct importance to the pigmentation per se [11]. 

Here, we investigate the structural integrity of the RPE in three different mouse models of RP (i.e., the rd9, Tvrm4, and rd10 mice) upon immunostaining for zonula occludens-1 (ZO-1), an essential component and regulatory element of RPE tight junctions [12]. All three models show a similarly abnormal RPE morphology with ZO-1 discontinuities and large interruptions, which temporally and topographically accompany the progressive demise of photoreceptors. Permeability assay by intravenous administration of non-permeant molecules demonstrates leakage and loss of sealing properties of the outer RBB in the areas where ZO-1 discontinuities occur. Noticeably, general administration of dexamethasone to rd10 mice during the time window of maximum photoreceptor loss, and shown before to successfully rescue cones and cone mediated vision in this mutant, also reduces the number of discontinuities in the RPE. Hence, inflammation in RP might be involved in RPE remodeling in a feedback loop, and might constitute a therapeutic target with beneficial effects on both photoreceptors and the RPE.

## 2. Results

### 2.1. ZO-1 Distribution Is Similarly Altered in Different Mouse Models of RP

Tight junctions are transmembrane and peripheral cytoplasmic protein complexes, which seal neighboring cells forming barriers that limit paracellular diffusion. In the RPE, ZO-1 is the most expressed barrier protein, with both structural and regulatory functions. To investigate the morphological integrity of the outer BRB, we used immunocytochemistry (ICCH) to analyze qualitatively and quantitatively the distribution of ZO-1 in the RPE of three distinct animal models of RP, expressing different causative mutations of the disease and showing various degrees of photoreceptor degeneration. 

The morphological findings are illustrated below, together with a brief summary of the three mutant phenotypes.

**Figure 1:**
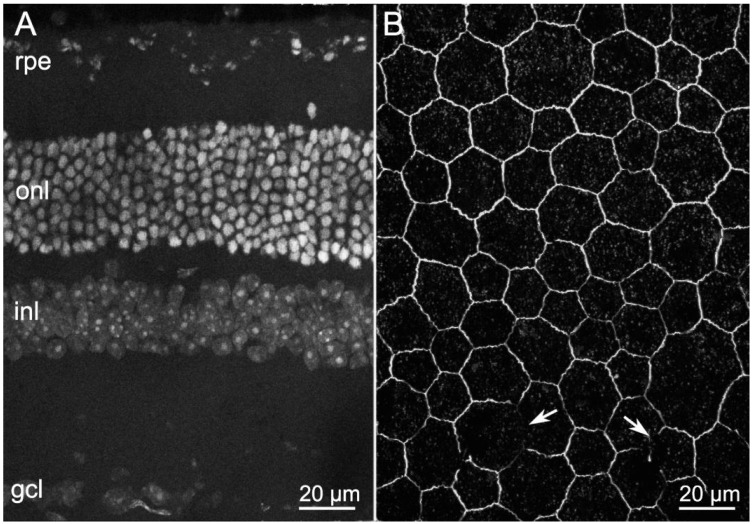
Retinal and RPE morphologies in wild type (wt) mice aged 1 year. (**A**) Retinal vertical section, DAPI nuclear staining. (**B**) Whole-mount RPE, ZO-1 immunostaining. Arrows show short and rare interruptions of the RPE (hexagonal) array. In this and in other Figures: onl—outer nuclear; inl—inner nuclear; and gcl—ganglion cell layer.

**Figure 2:**
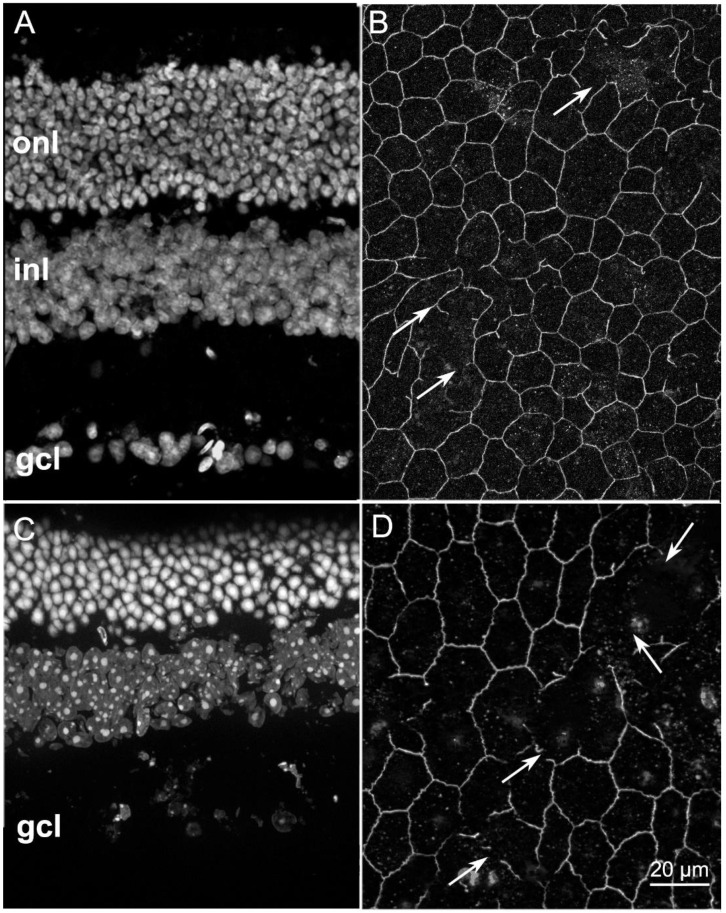
Retinal and RPE morphologies in rd9 mice aged 12 (**A**,**B**) and 20 months (**C**,**D**). (**A**,**C**) Retinal vertical sections. DAPI nuclear staining. Note the progressive thinning of the ONL from (**A**–**C**) and the poor retinal layering compared with the wt of Figure 1A. (**B**,**D**) ZO-1 staining of RPEs. Arrows point to large discontinuities, evident at 20 months (**D**). All images are from the central retina/RPE.

We studied RPE morphology of rd9 mice, which carry a spontaneous mutation in the retinitis pigmentosa GTPase regulator (*RPGR*) gene, located on the X chromosome and known to play a key role in the functions of the connecting cilium [13]. rd9 mutants are a rare model of X-linked RP, characterized by a slow phenotype, gradually progressing between 6 and 24 months of life and beyond [14]. As shown before [15], compared with the retina of a wild type (wt) mouse aged 1 year (Figure 1A), the outer nuclear layer (ONL) of aged-matched rd9 mice shows only a slightly lower number of irregularly arranged rows (Figure 2A) and yet an altered ERG indicative of photoreceptor loss [15]. Morphological alterations in the RPE are however readily appreciated upon examination of ZO-1 distribution and comparison to wt at the same age (Figure 1B and Figure 2B). The normal, hexagonal pattern of RPE cells highlighted by ZO-1 staining in the wt becomes evidently distorted in the rd9 counterpart and the occasional, short interruptions in the staining occurring in the first (arrows in 1B) become large and frequent in the second, while the labelled profiles are visibly thinner (arrows in 2B).

Older (20 months of age) rd9 mice show a further decrease in the number of rows of photoreceptor nuclei in the ONL, indicating retinal phenotype worsening (Figure 2C). The analysis of ZO-1 distribution in the RPE at this age shows visibly larger discontinuities (Figure 2D, arrows), suggesting progressive (and regressive) remodeling of the RPE. 

**Figure 3:**
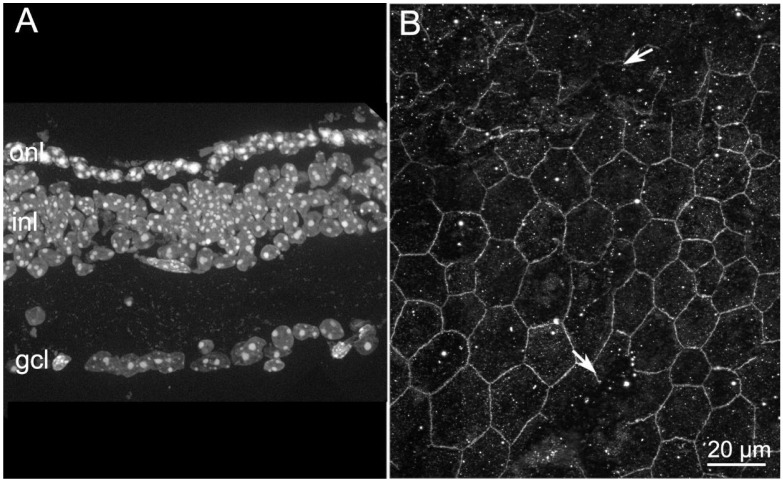
Retinal and RPE morphologies in rd10 mice aged 45 days. (**A**) DAPI nuclear staining of retinal vertical sections. Note the persistence of only one row of nuclei in the ONL. (**B**) ZO-1 staining of RPE from a similar 45 day old mouse in which several wide discontinuities can be appreciated (arrows). Both images are obtained from the central retina/RPE.

We extended the RPE analysis to rd10 mutant mice, which carry a missense mutation (R560C) in exon 13 of the beta subunit of the phosphodiesterase (*Pde6b)* gene. Retinal histology shows progressive ONL degeneration starting from the central retina at around 18 days of age (P18), with a fast wave of rod photoreceptor death peaking at around P25, followed by a delayed phase of cone death reaching a maximum at P45–P50 [16]. By P60, both rods and cones have largely died out and the ERG is almost extinct. This well-known model of autosomal recessive RP has been widely used for retinal rescue studies. Here, we investigated RPE organization at P45, when rod death is largely complete and cones and residual vision are rapidly declining. At this stage, only one row of nuclei (mostly belonging to cones) persists in the ONL (Figure 3A). Examination of the RPE shows evident interruptions in ZO-1 staining (Figure 3B, arrows), similar to those observed in the RPE of rd9 mutants. A high background, unspecific fluorescence is also detectable. 

**Figure 4:**
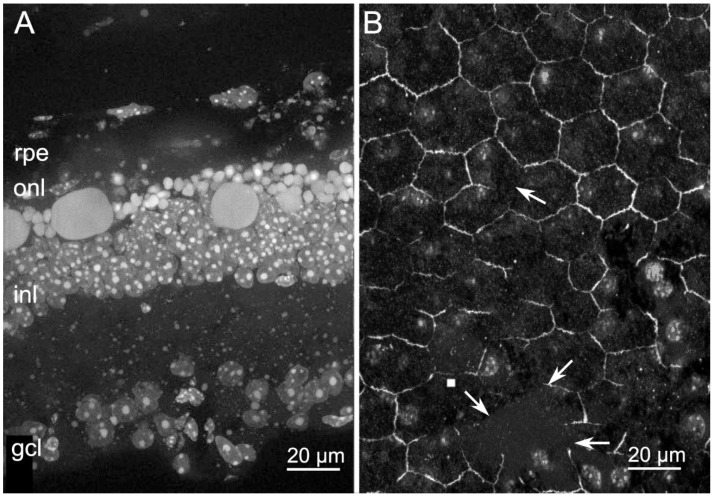
Retinal and RPE morphologies in light-induced Tvrm4 mice. (**A**) DAPI nuclear staining of retinal vertical sections from Tvrm4 mouse one week post light-induction. Note the presence of gigantic apoptotic bodies in the ONL, which is composed of few disorganized rows of nuclei compared with the wt. (**B**) ZO-1 staining of RPE from Tvrm4 mouse one week post light-induction. Arrows show wide discontinuities in ZO-1.

The analysis of the RPE morphology was extended to the Tvrm4 mutant mouse, which bears a dominant mutation (specifically, a substitution of isoleucine to asparagine at position 307, I307N) in the 7th transmembrane domain of the rhodopsin protein (RHO). As known, the mutation is virtually silent if mice are maintained in normal, ambient light levels but it is triggered by short exposures to very strong light (i.e., thousands of Lux), with subsequent retinal degeneration [17], irrespectively of age. In our experiments we consistently used mice aged between 1.5 and 5 months and used a consolidated light-induction protocol, known to affect the central retina only and leaving a ring of intact tissue at the periphery [18]. The RP phenotype is quite fast and photoreceptor loss 1 week post light-induction is evident (Figure 4A). 

Characteristic, gigantic apoptotic bodies, corresponding to DNA aggregates of dying photoreceptors, are visible in the ONL, where remaining nuclei stain intensely with DNA-binding molecules (Figure 4A). Correspondingly, the RPE abutting retinal areas of active photoreceptor degeneration shows an abnormal distribution of ZO-1: stained profiles are thin and characterized by large discontinuities (Figure 4B, arrows), which form wide islands from where ZO-1 is missing and conferring upon the RPE the appearance of a stretched net. Noticeably, the periphery of the eye, which is not reached by the inducing light, and where the retina does not degenerate, displays a correspondingly normal RPE, where ZO-1 resembles that of a normal, wt mouse, as shown later.

**Figure 5:**
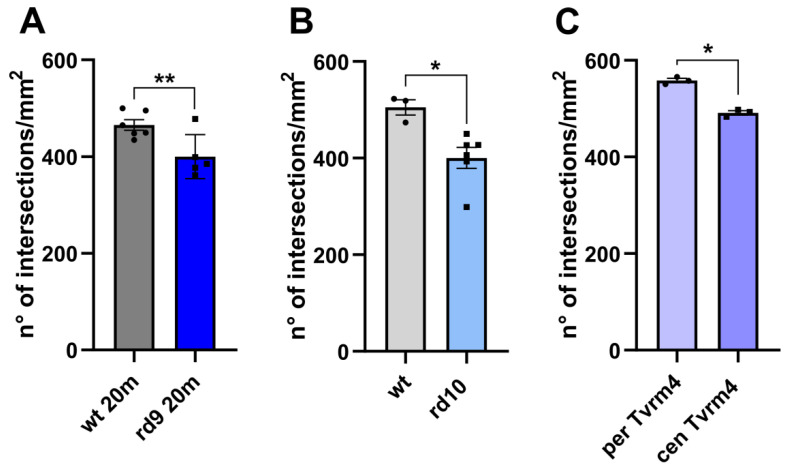
Density of ZO-1-positive profiles in the RPE of different mouse models of RP. (**A**) Comparison between 20 m old rd9 (n = 5) and age-matched wt mice (n = 6). Unpaired t test, *p* = 0.0158. (**B**) Comparison between rd10 (n = 6) and age-matched wt mice (n = 3) (all 45–50 days old). Unpaired t test, *p* = 0.0168. (**C**) Comparison between central (cen Tvrm4, n = 3) and peripheral (per Tvrm4, n = 3) zones of the RPE of Tvrm4 mice. Paired t test, *p* = 0.0138. Error bars represent ±SEM. * *p* < 0.05, ** *p* < 0.01.

The morphological observations of the RPE of three different mutants highlighted similar abnormalities, mainly represented by thinning and interruptions in ZO-1 staining. This prompted a quantitative estimation of the entity of these discontinuities and the subsequent search for a correlation to retinal phenotype severity. To this purpose, we carried out a quantitative analysis of the density of ZO-1-positive profiles, which were counted on microscope images of the RPE of the three mutants and of respective controls. Images were obtained at regular intervals on whole mount RPE samples and ZO-1-positive profiles counted with Image J and the corresponding data compared statistically as explained in Materials and Methods. Results are reported in Figure 5A–C.

All mutants show significant interruptions in the distribution of RPE-ZO-1 profiles (Figure 5). rd9 and rd10 mutants show a lower density of ZO-1 profiles in the RPE with respect to wt controls (Figure 5A,B). Tvrm4 mutants show a similar difference when central and peripheral RPE areas are compared (Figure 5C) (See also Supplementary Figure S1). As stated before, in the light-inducing conditions used here, Tvrm4 mutants develop a massive degeneration of the central retina only, with the peripheral ring remaining unaffected. The central-to-periphery pattern of degeneration proper of the retina of rd9 and rd10 mutants is milder, resulting in a lack of statistically relevant differences in local ZO-1 distribution in the RPE at the ages tested here (Supplementary Figure S2A–C). These findings suggest a direct connection between the primary photoreceptor degeneration responsible for RP and the observed remodeling of the RPE. 

**Figure 6:**
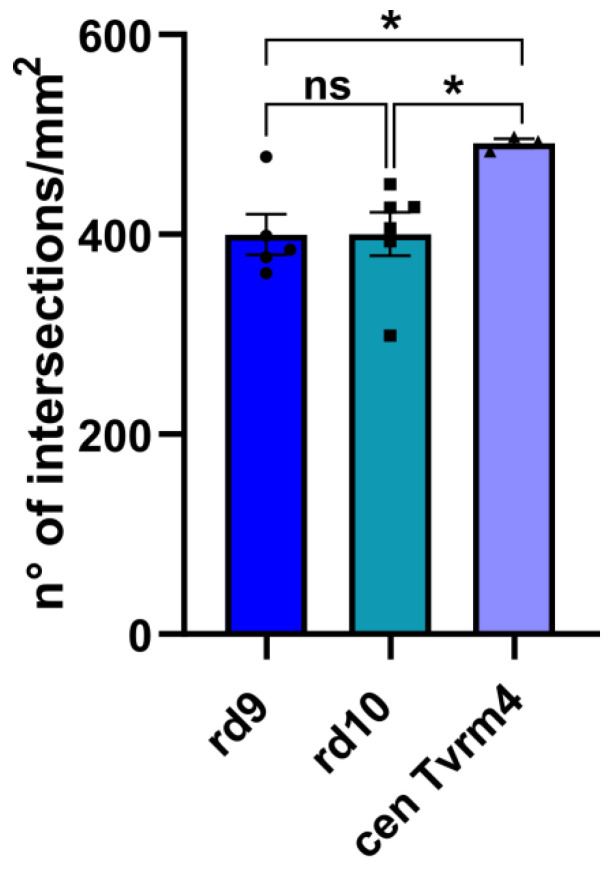
ZO-1 density distribution in the three mouse strains. Comparison among ZO-1 density profiles in the RPE of rd9 (n = 5), rd10 (n = 6), and Tvrm4 (n = 3, central RPE). One-way ANOVA *p* = 0.0327. Post hoc Tukey’s test. rd9 vs. rd10, *p* = 0.9999; rd9 vs. Tvrm4, *p* = 0.0457; rd10 vs. Tvrm4, *p* = 0.0400. Error bars represent ±SEM. * *p* < 0.05; ns: not significant.

We then searched for a correlation between the severity of retinal degeneration in the three mutants and the entity of RPE morphological abnormality, directly comparing ZO-1 densities in the three strains (Figure 6). We found a statistically significant difference between ZO-1 densities in rd9 and Tvrm4 mutants, as well as between rd10 and Tvrm4 mice. However, post hoc multiple comparisons showed similarities between the rd9 and rd10 pattern of ZO-1 interruption, despite the more pronounced retinal degeneration of rd10 mutants at the age shown here. Conversely, the analysis showed a better preservation of ZO-1 profiles in the RPE of Tvrm4 mice. 

**Figure 7:**
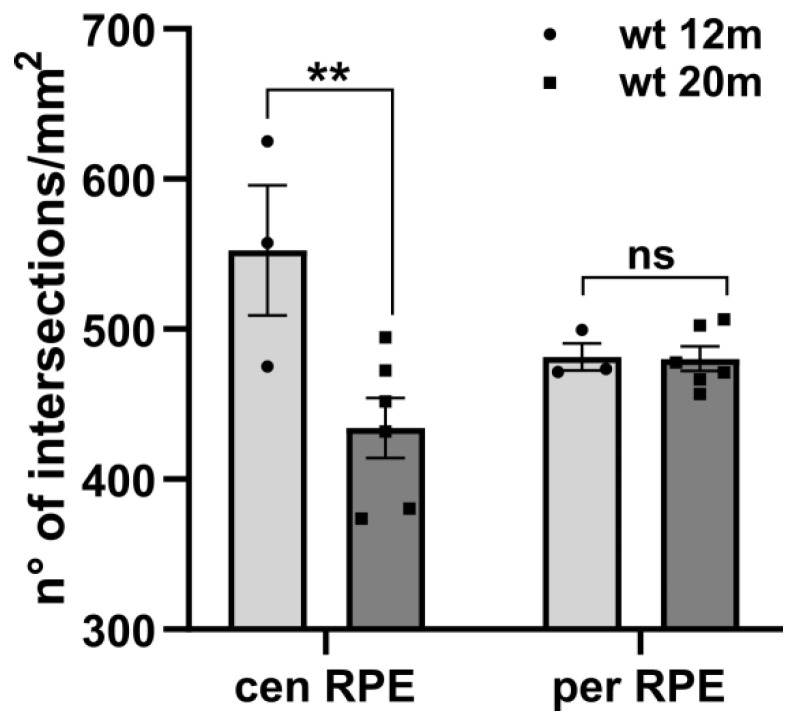
Aging contribution to changes of ZO-1 density. Comparison between RPEs of wt animals, aged 12 (n = 3) and 20 (n = 6) months, respectively, distinguishing central (cen RPE) and peripheral (per RPE). Two-way ANOVA *p* = 0.0162. Post hoc Sidak’s test. wt 12 m cen RPE vs. wt 20 m cen RPE, *p* = 0.0095; wt 12 m per RPE vs. wt 20 m per RPE, *p* > 0.999. Error bars represent ±SEM. ** *p* < 0.01; ns: not significant.

Knowing that RPE organization is naturally affected by aging [19,20], we tested the hypothesis that ZO-1 density could change with time as well, particularly affecting the density measurements of rd9 mutants, the oldest examined here. Hence, we compared ZO-1 data of wt mice aged 12 and 20 months; this analysis demonstrated a statistically significant decrease in ZO-1 density in the central (but not peripheral) RPE of older wt mice (Figure 7) confirming a contribution of aging to RPE remodeling. 

**Figure 8:**
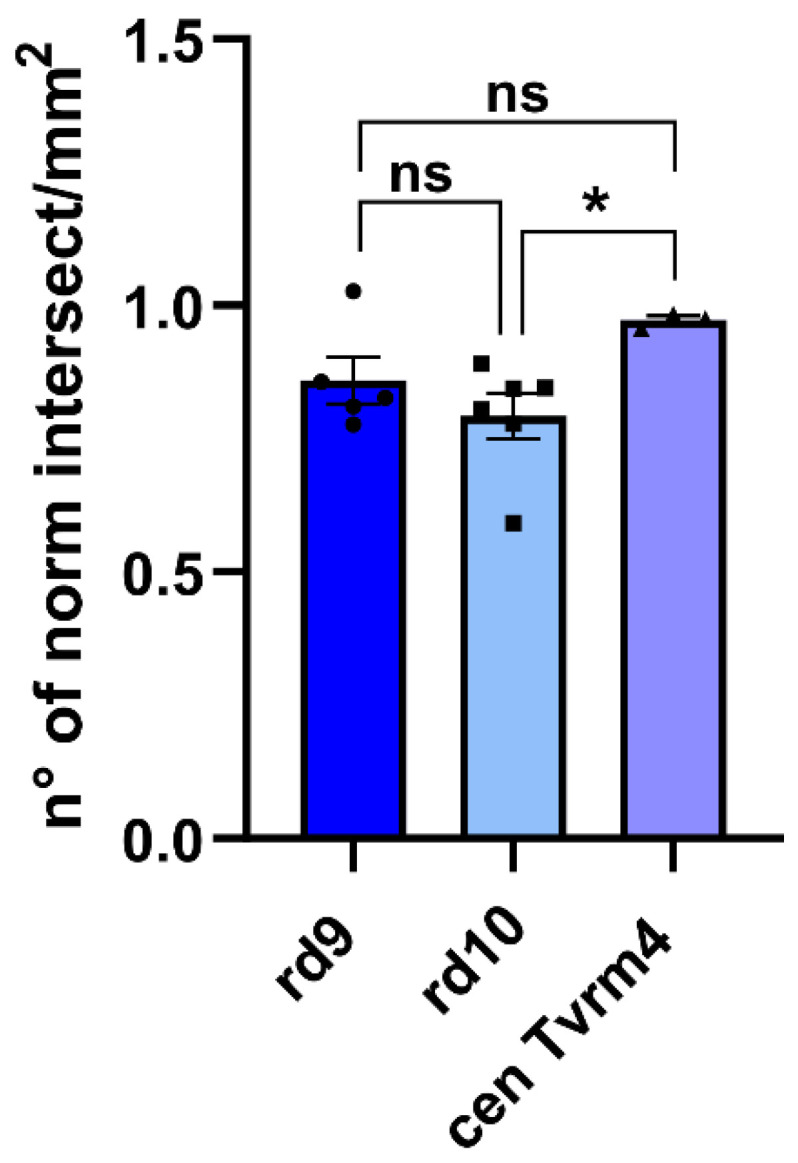
ZO-1 discontinuities are a stereotyped form of RPE remodeling. Comparison among the same groups of data used to generate Figure 5, each normalized to the respective control group (i.e., wt aged 20 months for rd9 mutants; wt aged 45–50 days for rd10 mice and for central Tvrm4 RPE). One-way ANOVA *p* = 0.0566. Post hoc Tukey’s test. rd9 vs. rd10, *p* = 0.479; rd9 vs. Tvrm4, *p* = 0.2614; rd10 vs. Tvrm4, *p* = 0.0465. Error bars represent ±SEM. * *p* < 0.05; ns: not significant.

A direct comparison between ZO-1 density data from the three genotypes, normalized to respective controls (namely: wt aged 20 months for rd9 mutants; wt aged 45–50 days for both rd10 mice and Tvrm4, central RPE) reduced age-dependent disparities (Figure 8); yet a slightly higher preservation of Tvrm4 RPE, as opposed to a higher degree of remodeling found in rd10 mutants, indicated some mutation-dependent effect. 

Hence, ZO-1 discontinuities can be considered a stereotyped form of RPE remodeling in RP, irrespectively of the genetic defect, the modality of transmission and the entity of photoreceptor loss; their extent is likely influenced by mutation and phenotype stage, in a yet to be established manner.

### 2.2. Permeability Assay of the Outer BRB Demonstrates Leakage

**Figure 9:**
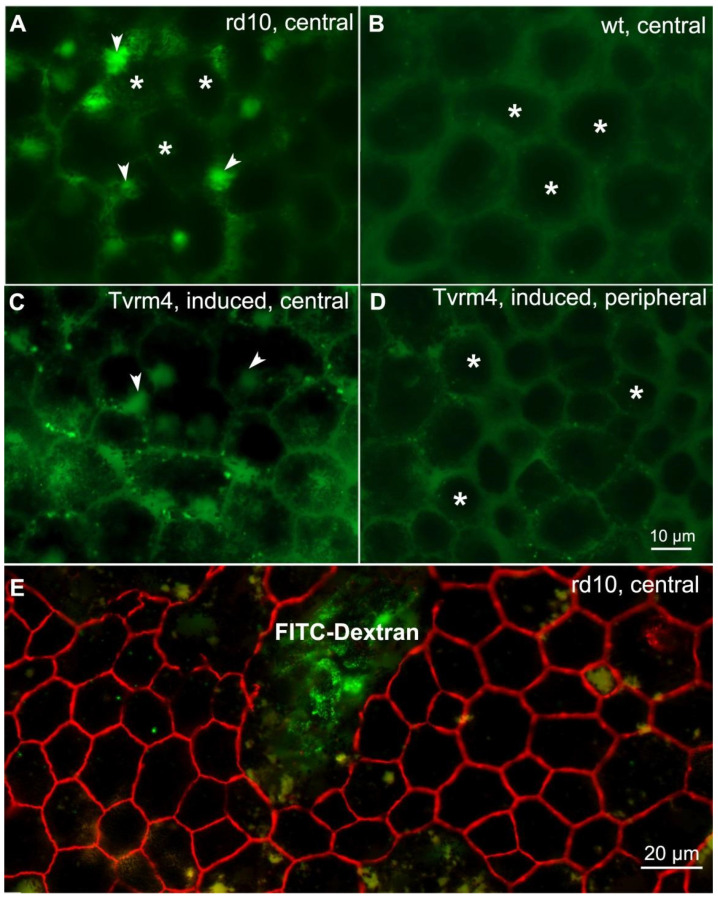
Functional and structural breakdown of the outer BRB. Whole-mount RPEs from rd10 (**A**,**E**), wt (**B**) and light-induced (**C**,**D**) Tvrm4 mice after intravenous injection of FITC-Dextran. Arrowheads show leakage of the fluorescent probe from choroidal vessels between the RPE cells (asterisks). Leakage of the bright, green-fluorescent probe occurs only in mutant mice (**A**,**C**), showing that the wt RPE (**B**) and the peripheral RPE of Tvrm4 mice (**D**) are intact. E—ZO-1 immunostaining (red signal) and FITC-Dextran (green) in the central RPE portion (rd10 mutant). A large ZO-1 discontinuity overlaps the area of main leakage.

To investigate the functional effects of the ZO-1 discontinuities observed in the RPE of RP mice, we performed an in vivo permeability assay. Dextran, a glucose polymer available in different molecular weights, conjugated with fluorescein isothiocyanate (FlTC), has long been used as a probe for vascular permeability studies [21]. Permeability of the FITC-dextran probes through the choroid-RPE is known to fall when increasing the size of the probes. High molecular weight dextranes, injected in the bloodstream, reach the extravascular space of the choroid through fenestrated capillaries but are arrested by the RPE tight junctions, which limit their permeation to the retina. Suspecting a major leakage of the outer BRB following the stereotyped finding of ZO-1 interruptions in our RPE preparations, we injected FITC-Dextran of 2000 kDa in the tail vein of rd10, Tvrm4 (light-induced) and wt mice, all aged 3–4 months. These mutants undergo RPE remodeling similar to that of rd9 counterparts, but on a faster temporal scale, and therefore were chosen for acute experiments. Results from RPE fluorescence microscopy observations are summarized in Figure 9. 

As predictable, preparations from rd10 and Tvrm4 mutants show evident leakage of FITC-Dextran from adjacent choriocapillaris through the RPE (Figure 8); the green fluorescence signal concentrates at the apical side of the RPE, in the subretinal space, forming bright clusters (Figure 9A). Leakage of the fluorescent probe is neither observed in the RPE of wt animals (Figure 9B) nor from the intact, peripheral region of the RPE of light-induced Tvrm4 mice (Figure 9D), while it is evident in their central portions (Figure 9C). Co-staining with antibodies against ZO-1 shows that large zones of dextran penetration correspond to equally extended areas of discontinuity in ZO-1 (Figure 9E), demonstrating that this morphological abnormality is indicative of major leakage of the outer BRB.

### 2.3. Abnormal RPE Vacuolization in Tvrm4 Mutants

**Figure 10:**
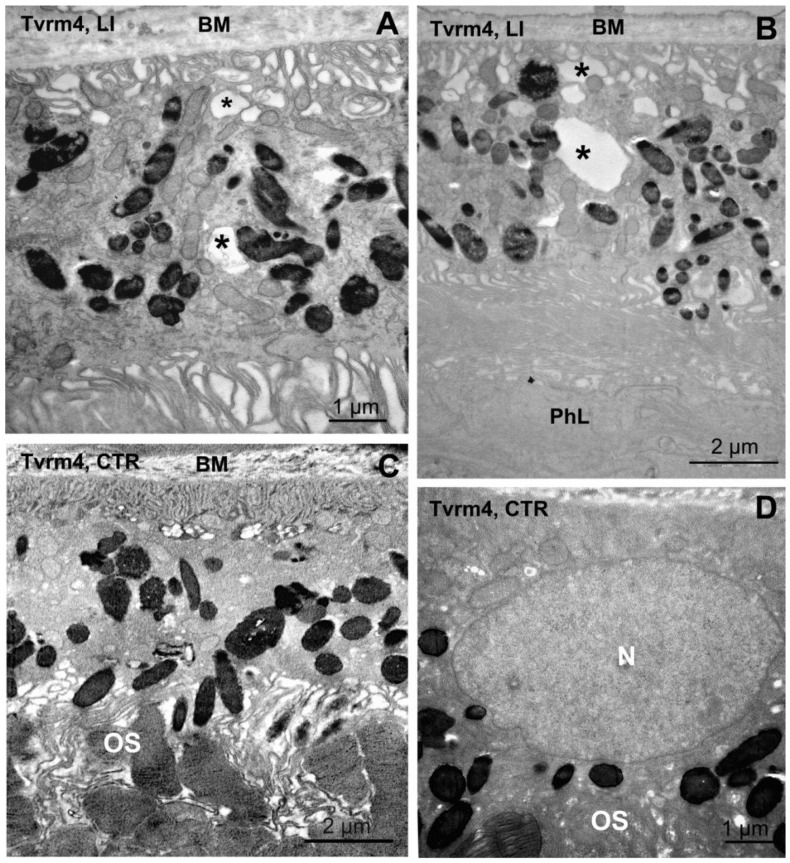
Vacuolization of RPE cells. Ocular vertical section from Tvrm4 mice, 4 weeks following light-induction (**A**,**B**) and in a non-induced littermate (**C**,**D**). Transmission electron microscopy shows membranous vacuoles which accumulate at the basal side (abutting Bruch’s membrane, BM) in the proximity of the basal infoldings of RPE cells of the light-induced samples (asterisks in (**A**,**B**)); the photoreceptor layer is absent or completely disorganized. In the non-induced control, outer segments (OS) are clearly visible. N—nucleus of RPE cell.

Preliminary electron microscopy examination was carried out on ocular samples previously prepared to study synaptic distribution in the central retina of light-induced, Tvrm4 mice, at a stage of total photoreceptor loss (4 weeks post induction) [22]. Ocular vertical sections included the retina (either degenerating or intact) and the overlying RPE, choroid, and sclera. EM analysis showed that RPE leaflets abutting points of complete photoreceptor degeneration appeared filled with numerous membranous vacuoles, with minimal ultrastructural details and of various size but invariably concentrated at the basal side of the RPE, in the proximity of Bruch’s membrane. At times, these cytoplasmic vacuoles were large but still with minimal electron density (Figure 10A,B, asterisks). Vacuoles were much rarer in RPE areas of non-induced, control eyes, showing still intact photoreceptors (Figure 10C,D). Although preliminary, the observation that cytoplasmic vacuoles build up on the basal RPE side is worth reporting for their resemblance to membrane-bound inclusions described in the RPE of various models of ocular diseases and particularly of macular degeneration. Noticeably, large membrane-bound vacuoles have been observed to overlie drusen deposits in eyes of MD donors [23]. These vacuoles likely belong to a recurrent repertoire of RPE anomalies common to different pathological conditions and indicative of abnormal, sustained transit of fluids through the RPE. A systematic ultrastructural analysis of the RPE of Tvrm4 mutants is in progress for future studies.

### 2.4. Dexamethasone Administration to rd10 Mutant Mice Rescues ZO-1 Density

Alterations of brain and retinal blood barriers occur in pathological conditions and are a common finding of inflammatory diseases (i.e., diabetes). In a recent study [24], we demonstrated that dexamethasone treatment of rd10 mice reduced retinal inflammation in these mutants, achieving cone photoreceptor rescue. We wondered whether this treatment could influence the RPE as well, in particular reducing the entity of ZO-1 interruptions described here. 

**Figure 11:**
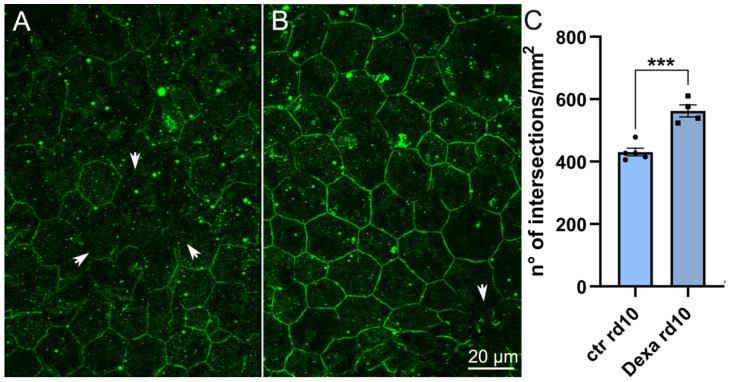
Whole-mount RPE stained for ZO-1 obtained from P45, rd10 mice administered with saline, control solution (**A**) and dexamethasone (**B**). ZO-1 interruptions (arrowheads) are visibly fewer in (**B**) and the density of ZO-1 stained profiles correspondingly higher (**C**). *** *p* < 0.001.

We exploited RPE samples collected from rd10 mice aged 45 days and treated with subcutaneous dexamethasone injections from P23. Retinal samples from these (and littermate control mice, injected with water) have been previously used to demonstrate a rescue effect of this steroid on retinal cones [24]. ZO-1 staining performed with the same criteria described above showed by direct examination with fluorescence microscopy that discontinuities were visibly rarer in the RPE of dexamethasone-administered mice (Figure 11A,B). Quantitative analysis confirmed this observation, demonstrating a higher density of ZO-1 profiles in the RPE of treated mice (Figure 11C). Thus, administration of dexamethasone to rd10 mutants lowers retinal inflammation, rescues cone structure and function, and limits the formation of discontinuities and barrier leakage of the RPE.

## 3. Discussion

In previous studies we demonstrated an almost 4-fold decrease in ZO-1 immunofluorescence in the RPE of rd9 mice aged 12-18 months compared with wt counterparts [15], suggestive of a structural weakening of junctional complexes. Here, we extend this observation showing a lack of continuity in the staining for ZO-1 in the RPE of the same mutant, also recurring in two additional models of RP. This morphological finding has an expected functional correlation, insofar as the outer retinal barrier demonstrates abnormal leakage of high molecular weight molecules in the same areas where ZO-1 staining is interrupted. We also report here the EM observation of extensive vacuolization at the basal side of RPE cells of Tvrm4 mutants exposed to light and undergoing profound photoreceptor loss. Similar vacuoles have been described in RPE samples of several disease models, as well as in the eyes of human AMD donors studied with high definition confocal analysis and/or electron microscopy [25]. We believe that vacuoles reported here closely resemble those of mice exposed to cigarette smoke [26], as well as ultrastructural inclusions found in the RPE of mice with abnormal choriocapillaris, mimicking geographic atrophy. In general, these vacuoles are regarded as places of abnormal fluid entry at the basal site of RPE cells, arising secondarily to oxidative stress known to build up in the subretinal space in AMD and exacerbated by cigarette smoke exposure [27]. Although not directly linked to the observation of oBRB leakage, primarily reported here, vacuoles in the basal RPE represent the recurrent component of a local inflammatory state, known to be associated with photoreceptor degeneration. Inflammatory molecules originating from the choroidal vessels could be absorbed through RPE membranous vacuoles further reinforcing inflammation. Noticeably, in cigarette-smoke-exposed mice, RPE vacuoles are mitigated by genetic ablation of CxCr5, a receptor that plays a basal role in B cells migration and local inflammation/immune response [28].

These data bring to light the occurrence of stereotyped (and previously unreported) abnormalities in the RPE of different mouse models of RP, representing as many paradigms of this heterogeneous family of disorders, also indicative of three different modalities of inheritance transmission. Remarkably, the entity of ZO-1 interruption (indicative of barrier breakdown) does not correlate entirely with the degree of photoreceptor loss, appearing as a partially mutation-independent event. And yet, there is a strict topographical correlation between retinal degeneration and formation of ZO-1 discontinuities. They also become more frequent in the process of physiological aging, possibly as a consequence of time-dependent accumulation of oxidative stress and inflammation [20,29]. Time-dependence would explain why ZO-1 interruptions are not as frequent as expected in an abrupt paradigm of photoreceptor degeneration such as the one of Tvrm4 mutants studied here. Despite the rapid loss of rods and cones form the central retina, secondary, bystander effects, related to oxidative stress and inflammation becoming chronic, did not have enough time to build up and negatively affect RPE cells in the short post-induction time window used here. 

Tight junctions are complex structures, known to include over 40 proteins, broadly comprising transmembrane constituents and intracellular, scaffolding proteins. Among the latter, ZO-1 anchors the macromolecular complexes of tight junctions to cytoplasmic actin; its disruption results in direct loss of barrier function and a reorganization of the cytoskeleton [3,30,31]. Abnormalities like the ones reported here and the creation of interruptions in ZO-1 array have been described in AMD, both in human patients and multiple experimental models, either based on light damage or choroidal neovascularization. Tight junctions alone have been exploited as targets for developing therapies for AMD and an impressive body of literature illustrates oBRB abnormalities occurring in this disease and in corresponding experimental models, addressing cellular and molecular mechanisms underlying the disease outcome (as an example, see [7,9,30,32,33,34,35,36]). Tight junction impairments are also documented in other retinal pathologies with a strong inflammatory component (i.e., typically, diabetic retinopathy, as well as macular edema and uveitis) [37]. When the outer BRB breaks down, anomalous entry of proteins (that comprise inflammatory species), ions, and fluids is allowed from the choriocapillaris versus the subretinal space [38], favoring protein precipitation, accumulation, and possibly edema [33]. Similar effects are likely to occur in RP as well but have received correspondingly minor attention, possibly because of the lower incidence of RP compared with other diseases and because photoreceptor degeneration of genetic origin is the primary and prevailing feature of RP. 

Abnormalities in the RPE have already been reported for Tvrm4, light-induced mice [39]; specifically, the authors described interruptions in ZO-1 and a translocation of alpha-catenin from cytosol to nucleus of RPE cells, which were also found to exhibit abnormal shape and size. Alpha-catenin is a mechanosensor responsible for integrity of epithelial cells as well as an inhibitor of transcription, decreasing Wnt/β-cat–responsive genes expression. In turn, this pathway is responsible for induction and maintenance of the blood brain barrier through transcriptional regulation of junctional proteins, suggesting a similar role in RPE cells. ZO-1 displays a similarly dual role: it is a structural and functional, tight junction protein, which can also control the activation of the transcription factor ZONAB. Downregulation of ZO-1 and upregulation of ZONAB nuclear activity promote RPE cell proliferation [40,41] and alter RPE morphology. All these molecules are good candidates to explain the mechanisms of RPE structural remodeling observed here and deserve further investigation. Yet, it remains to be understood which molecular signals trigger RPE remodeling, albeit for RP this process is likely to be initiated by photoreceptor death. A possible mechanism linking the two processes has been suggested in a recent paper [42], showing that photoreceptor death greatly reduces the need of RPE cells to phagocyte their outer segments; in turn, this leads to inhibition of the Akt pathway in RPE cells, and consequent reduction of glucose transport from the RPE to photoreceptors. This contributes to cone starvation and death, which further feed metabolic and structural changes in the RPE. 

Additional links between inherited photoreceptor degeneration and RPE functional abnormalities might be searched in the pathogenesis of RP, in which oxidative stress and inflammation contribute to the bystander death of cones. RPE cells, with their intimate structural and metabolic association to photoreceptors, are expected to suffer from similar, bystander consequences. High levels of non-consumed oxygen are responsible for increasing the fraction of free ROS in the outer retina [43,44]. Functionally, unbalanced ROS can damage membrane lipids, nucleic acids, and proteins, including epithelial tight junctions [45]. Hence, the observed reduction of ZO-1 staining reported here could be due, at least in part, to the oxidative environment surrounding RPE cells during photoreceptor degeneration. In addition, the concomitant process of inflammation/immune response leads to local release of active molecules (chemokine, cytokines) and enzymes (metalloproteases, MMPs) which can further compromise RPE integrity. In vitro studies demonstrated that TNFα (a common inflammatory cytokine) activates the transcription factor NF-κB and decreases the levels of ZO-1 in RPE cell lines; in vivo, pharmacological inhibition of this pathway rescues the integrity of tight junction complexes in a mouse model of diabetic retinopathy [46]. Mouse RPE explants incubated with activated neutrophils show reduced expression of ZO-1; the effect is partially mediated by MMP9, whose inhibition attenuates the reduction of ZO-1 caused by neutrophil exposure [47,48]. Finally, accumulation of macrophages in the outer retina receives a contribution from choroidal infiltrates (reviewed in [49]), as the choroid hosts macrophages, dendritic cells, and mast cells. Passage of molecules from these cells is likely to be facilitated by the abnormal leakage of the oBRB observed here. This process, in turn, reinforces the general inflammatory reaction taking place in the outer retina, further supporting bystander aggression of cones and RPE, in a classical vicious cycle. 

Dexamethasone might alleviate this process directly acting on glucocorticoid receptors, whose presence has been shown in human RPE, albeit not yet in mice; or indirectly, by decreasing cone death as previously shown [24]. Probing this drug on RP might be facilitated by its current employment to treat cystoid macular edema, a classical complication of this disease [50,51]. Altogether, rearrangements in the RPE of RP mice are similar to abnormalities shown in AMD, suggesting the possibility to also evaluate the therapeutic value of this important organ for treating inherited retinal degeneration.

## 4. Materials and Methods

**Animal procedures**

Animal experimental procedures were approved according to the current regulations as reported in the Institutional Review Board Statement. A minimum of n = 3 animals were used per experimental group. All mice were originally from Jackson (Bar Harbor, ME, USA). They were maintained and bred in a local animal house with water and food ad libitum, in a 12 hrs dark/light cycle and illumination levels below 100 Lux. Mice of the C57Bl6J wild type (wt) strain were used for controls. They were harvested at 3–4 (young adults), 12 and 20 months. A total of 12 wt mice were used for the study.

**Mice**

Naturally occurring rd9 mutants on a C57Bl6/J background were also used. Specifically, the RPE from ocular samples of male rd9/Y and female rd9/X mice used in a previous retinal study [15] were examined here, together with the RPE of additional rd9 mutants. A total of 6 rd9 mice were used for quantitative analysis.

Heterozygous (HT) Tvrm4 mice (RhoTvrm4/Rho+) on a C57Bl6J background, aged 3–4 months, were dark adapted for 4 hrs in a black cage and then administered eye drops (1 µL of 0.5% atropine, Allergan). Afterwards, mice were placed in a black illumination chamber described before [18] equipped with fluorescent bulbs (Philips Master TL5 HE 28 W/840, length 115 cm; 104 lm/W cool white mercury lamps) and exposed for 2 min to a 12,000 Lux intensity. Mice were then returned to their cages, in normal animal house illumination conditions and harvested 1 week post induction (PI) to obtain ocular samples. A total of 6 Tvrm4 mice were used.

Homozygous rd10 mice (Pde6b^rdl0/rd10^) on a C57Bl6J background, aged P45-P50 were also used (n = 4) for RPE morphological studies.

**RPE and retinal preparation**

Mice were deeply anesthetized by intraperitoneal injections of Zoletil 100 (80 mg/kg) and their eyes quickly removed. Mice were then killed by cervical dislocation or decapitation. Enucleated eyes were submerged in buffer and opened to remove the anterior segment; 4 radial incisures were cut toward the optic nerve head and the retina was gently separated and processed independently. The sclera-choroid and RPE were further cut radially with additional 4 to 8 incisions and processed for whole mount immunocytochemistry (ICCH). After a block solution in 5% serum and 0.3% Triton-X 100 for 4 hrs, samples were incubated in rat polyclonal antibodies against ZO-1 (MABT11, Merck-KGaA, Darmstadt, Germany) diluted 1:400, for 4 days, at 4 °C. After rinsing in buffer, specimens were incubated in secondary antibodies (anti-rat IgG, conjugated with Alexa Fluor 488 or Rhodamine Red X, all from Invitrogen, Termofisher Scientific, Waltham, MA USA). After repeated washing, the RPEs were counterstained with Hoechst (0.02 mg/mL), flat mounted on glass slides and covered with Vectashield antifade mounting medium (Vector, Burlingame, USA). Additional eyes were fixed as above, dissected to obtain eye cups, infiltrated with 30% sucrose, snap frozen in isopentane/dry ice, embedded in Tissue-Tek OCT (Sakura Finetek Europe, B.V. Alphen aan den Rijn, The Netherlands), and stored at −20 °C. They were used for cryostat sectioning and nuclear fluorescent staining to visualize retinal layers.

**Imaging**

Images of RPE preparations were obtained with a Zeiss Imager.Z2 microscope equipped with an Apotome2 device (Zeiss, Milan, Italy), using a Plan Neofluar 40X, 1.25 oil objective. ZO-1-stained preparations were sampled regularly along 4 radial axes; along each radius, 1 central and 2 mid-peripheral sampling fields were selected with respect to the optic nerve emergence site, choosing optimally flattened areas. Hence, for each RPE specimen, 10–14 sampling fields, measuring (224 × 168) µm, were imaged. Sample images were obtained as z stacks encompassing the complete width of ZO-1 labelling as well as RPE nuclei and typically comprising 10–15, regularly spaced, sections. The Zeiss software ZEN^®^2 was used to adjust brightness and contrast of the pictures and to generate projection images which were saved as tiff files. Image J was used to count in each projection the number of intersections between ZO-1-positive profiles and an overlapping (custom-generated) grid, with 20 µm-spaced mashes. Tiled images of RPEs and corresponding retinal leaflets were also obtained at low magnification and stored. Image J data were analyzed statistically with GraphPad 8.0.2.

**Fluorescent dextran permeability assay**

To assess the capability of the outer BRB to properly exclude from the retina non-permeable tracers inoculated in the bloodstream, we injected in the tail vein 150–200 microliters of fluorescein isothiocyanate–dextran (FITC–Dextran 2000 kDa mw, Sigma, (FD2000S-100MG Merck-KGaA, Darmstadt, Germany) diluted in saline solution to have a final dose of 0.5 mg/k. Injections were performed on wt, rd10, and light-induced Tvrm4 mice (n = 3 per group). Their eyes were enucleated 30 min after the injection under deep general anesthesia and the animals killed afterwards by cervical dislocation or decapitation. Their eyes were dissected immediately, briefly (5 min) fixed in 4% PAF and used to prepare whole mount RPEs and retinas for fluorescence microscopy imaging of FITC-dextrans. Afterwards, RPEs (comprising the sclera and choroid) were processed for ZO-1 immunostaining, revealed using Alexa 568-conjugated secondary antibodies (Invitrogen, Termofisher Scientific, Waltham, MA, USA). Some eyes were quickly frozen and stored.

**Dexamethasone administration**

The RPEs of a group of rd10 mice previously used to study the effect of subcutaneous dexamethasone administration on photoreceptor survival, as described in [24], were examined here to assess the outcome of the pharmacological treatment upon the distribution of ZO-1 in the RPE. A total of 4 treated and 3 control rd10 mice aged 45 days were used for this study. Dexamethasone (Soldesam Forte 4 mg/mL; Laboratorio Torino Medica, Turin, Italy) or vehicle (an identical amount of distilled water) was administered daily (between 8 and 9 am) from P23 to P45 at a dose of 4 mg/kg. Eyes were enucleated as described above and the RPEs processed for ZO-1 ICCH.

**Statistical analysis**

All data were analyzed using GraphPad Prism 8.0.2. ZO-1 density quantification was performed using 3-6 RPE preparations from different animals per experimental group. Microscopy images were obtained from 12–16 fields (224 × 168) µm, for each RPE sample. Counting data (representing the intersections of ZO-1 profiles with a reference grid overlapping each image) were obtained with Image J, saved in Excel files, used to calculate ZO-1 density/mm^2^, and transferred to GraphPad for further analysis. Data were shown as mean ± standard error of mean (SEM), where each mean is obtained from 1 mouse. Statistical comparisons between mutant genotypes and the respective controls were conducted by means of two-tailed, unpaired, or paired Student’s t-test. Comparisons of means between all three mutants were determined using one-way ANOVA followed by Tukey’s post hoc test. Statistical differences between wt animals were tested for significance using two-way ANOVA considering age and RPE (central or peripheral) positions as variables. Two-way ANOVA was followed by Sidak’s post hoc test. *p* < 0.05 was considered to indicate a statistically significant difference. In the graphs, ns—not significative, * *p* < 0.05, ** *p* < 0.01 and *** *p* < 0.001.

**Electron microscopy**

Tvrm4 mice (n = 2) were light-induced (2’, 12,000 Lux) and after 28 days they were anesthetized as described above and their eyes rapidly enucleated. Eyes were also obtained from (n = 2) non-light-induced littermates. Eye cup fixation and embedding were as described in [22]. Briefly, eye cups were fixed in 1% glutaraldehyde-2% vol/vol PFA in Sorenson’s buffer M/30 (pH 7.4 + 5% wt/vol sucrose) for 2 h at 4 °C, dissected in quadrants and postfixed in 3% for 12 h, at 4 °C. Tissue blocks were stained with 3% potassium ferrocyanide +2% OsO4 in H2O, at 4 °C; bloc stained with 1% uranyl acetate (in 0.05 M maleate buffer, pH 6), then dehydrated with a graded series of ethanol and flat embedded in Epon/Araldite. Ultrathin sections, 90 nm thick, were obtained from the central retinas of light-induced and control samples, collected on single hole, formvar-coated grids, and examined with a Jeol 1200EXII electron microscope. Images were obtained at various magnifications using a Gatan high resolution cooled camera.

## Data Availability

Data supporting reported results can be requested from the corresponding authors; they are presently stored in a laboratory-owned archive to be released in the future.

## Supplementary Materials

The following are available online at https://www.mdpi.com/article/10.3390/ijms22105381/s1.
