# Supplementary material for: Retinal Pigment Epithelium Remodeling in Mouse Models of Retinitis Pigmentosa
Source: Int J Mol Sci. 2021 May 20;22(10):5381. doi: 10.3390/ijms22105381 (PMC8161377; doi:10.3390/ijms22105381)
Supplement: Supplementary file 1 [file ijms-22-05381-s001.zip › ijms-1217747-SI.pdf]

The following are available online at https://www.mdpi.com/article/10.3390/ijms22105381/s1

Figure S1: Density of ZO-1-positive profiles in the RPE of different mouse models of RP. Single counts distribution

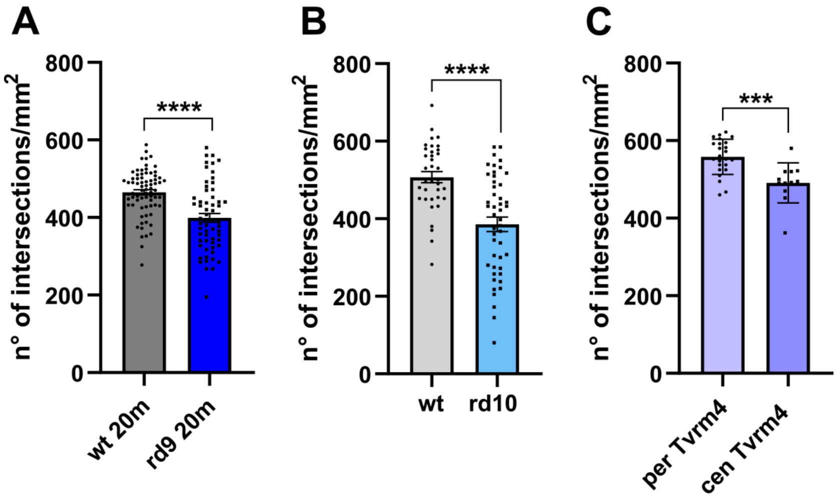

Supplementary Figure S1. Density of ZO-1 positive profiles in the RPE of the three different mouse models of RP. Single point representations: each dot represents a microscopic field of whole mount RPE used for counting. A. Comparison between 20 m old rd9 (n=61) and age-matched wt mice (n=74). Unpaired t test, p< 0.0001 B. Comparison between rd10 (n=48) and age-matched wt (n=36) (all 45-50 days old). Unpaired t test, *** p<0.0001. C. Comparison between central (cen Tvrm4, n=12) and peripheral (per Tvrm4, n=24) zones of the RPE of Tvrm4 mice. Paired t test, p= 0.0003. Error bars represent ±SEM

The following are available online at https://www.mdpi.com/article/10.3390/ijms22105381/s1

Figure S2: ZO-1-density distribution in control conditions for the three mouse strains under study

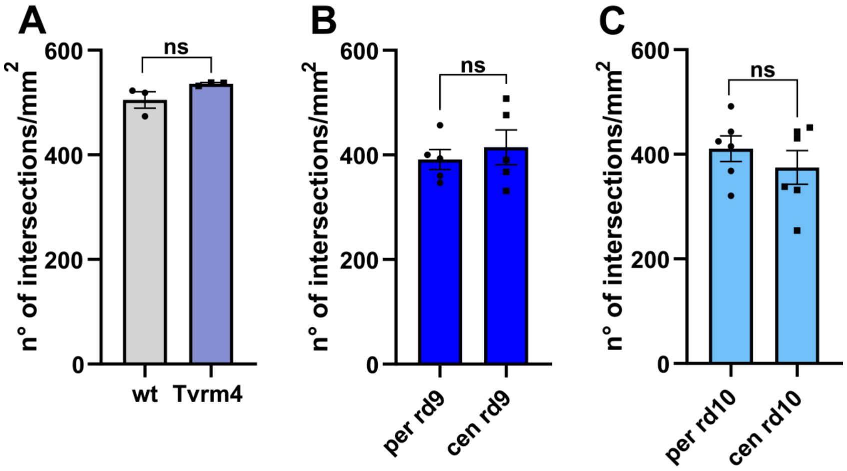

Supplementary Figure S2. A. Comparison between Tvrm4 (n=3) and age-matched wt (n=3). Unpaired t test, p=0.1230. **B.** Comparison between central (cen rd9, n=5) and peripheral (per rd9, n=5) zones of the RPE of rd9 mice. Paired t test, p= 0.5101**. C.** Comparison between central (cen rd10, n=6) and peripheral (per rd10, n=6) zones of the RPE of rd10 mice. Paired t test, p= 0.3697. Error bars represent ±SEM. ns: not significant.
